# Development of an Early Warning System for Owners Using a Validated Health-Related Quality of Life (HRQL) Instrument for Companion Animals and Its Use in a Large Cohort of Dogs

**DOI:** 10.3389/fvets.2020.575795

**Published:** 2020-09-24

**Authors:** Vinny Davies, E. Marian Scott, M. Lesley Wiseman-Orr, Andrea K. Wright, Jacqueline Reid

**Affiliations:** ^1^School of Computing Science, University of Glasgow, Glasgow, United Kingdom; ^2^School of Mathematics and Statistics, University of Glasgow, Glasgow, United Kingdom; ^3^School of Education, University of Glasgow, Glasgow, United Kingdom; ^4^Outcomes Research, International Centre of Excellence, Zoetis, Dublin, Ireland; ^5^NewMetrica Ltd., Glasgow, United Kingdom; ^6^School of Veterinary Medicine, University of Glasgow, Glasgow, United Kingdom

**Keywords:** Health-Related Quality of Life (HRQL), owner questionnaire, smartphone app, dogs, early warning system, wellness, preventive medicine

## Abstract

Preventive measures in human healthcare are recognized as a means of providing early detection of disease, however, the veterinary profession has not been as effective in communicating the benefits of preventive measures to pet owners. Readily available pet healthcare information on the internet, owners not understanding that regular health evaluations can ensure the well-being of their pets and owners confusing the signs of chronic disease with normal aging have contributed to declining numbers of veterinary visits. The use of web-based generic health–related quality of life (HRQL) measures to evaluate health status (wellness) remotely could facilitate veterinary preventive medicine. This publication describes the development and practical application of an integrated alert system for an online generic HRQL measurement instrument (VetMetrica™) which generates scores in four domains of HRQL—Energetic/Enthusiastic (E/E), Happy/Content (H/C), Active/Comfortable (A/C), and Calm/Relaxed (C/R)—for 2 age groups (young/middle-aged, ≤7 years and old, ≥8 years). The alert provides an early warning, via email to owners, that a potentially significant deterioration in health status has occurred. The model accurately predicted the health status of 93 and 83% of sick young/middle aged and old dogs respectively, with healthy dogs predicted with 83% accuracy. HRQL data, collected via a white-labeled veterinary clinic branded app designed to facilitate connected care between owner and veterinarian, were analyzed for 6,108 dogs, aged between 6 weeks and 16 years. Of these 5,002 were deemed to be in perfect health by their owners, yet the alert was triggered for 1,343 (27%) of these, 75% of which were young/middle-aged and 25% were old, indicating that acute injuries notwithstanding, many middle aged dogs may have been suffering from undetected chronic disease such as osteoarthritis. This work has demonstrated that the use of VetMetrica™ delivered via the PetDialog™ app, which supports 24/7 remote health monitoring is an efficient way for vets to provide all their owners with the opportunity to monitor their animal's wellness throughout their lifetime, providing the vet with a mechanism to identify health problems early while stimulating owners to be more proactive in seeking veterinary attention.

## Introduction

Preventive medicine in the food animal sector plays an important role in preventing and controlling important diseases of food animals and humans and involves a number of disciplines including epidemiology and public health. Similarly, in human healthcare preventive measures have long been recognized as a means of providing early detection of disease or avoiding it completely. In that regard, recent research developments highlight the value of Patient–Generated Health Information (PGHI), described as data created and recorded, by patients, family or personal caregivers, whereby health data can be shared with a health care provider via a smartphone health app or a patient portal accessed in the home (1). In human healthcare, patient symptoms, health status, and quality of life are increasingly monitored in this way. Receiving information directly from the patient or caregiver can be valuable in many ways. It can strengthen the patient/caregiver-clinician relationship, providing opportunities for shared decision making as well as capturing data that otherwise would be missed by traditional means. However, veterinarians working with companion animals have not been as effective in communicating these benefits of preventive measures to pet owners and this has contributed to a decade long trend toward declining numbers of pet owner visits to veterinarians. Even though the number of cats and dogs has increased and continues to do so, the footfall in veterinary practice has declined. In the US alone the cat and dog population increased >36% in the 10 years prior to 2006, yet American Animal Hospital Association data show that between 2001 and 2009 there was a decrease of 17% in the median number of active clients seen by full-time veterinarians (2). According to the 2011 Bayer Veterinary Care Usage Study this decline can, in part, be attributed to the readily available pet healthcare information on the internet, which for many owners is their first port of call when their pets show signs of illness. At best, this results in a delayed veterinary visit when symptoms persist, but may represent a missed visit if the animal improves. Perhaps more worrying is the fact that another major reason for the decline is lack of understanding on the part of pet owners that regular health evaluations will help ensure the well-being of their pets. Unfortunately, many pet owners still associate veterinary care primarily with vaccinations and the treatment of acute diseases or injuries, tending to confuse the signs of chronic illness with the aging process. Furthermore, with the increase in the companion animal population has come a disproportionate increase in the number of geriatric animals and a consequent increase in the incidence of chronic disease. Indeed according to the Banfield Pet Hospital, state of pet health reports (3–5), there has been a substantial increase in the incidence of diabetes and obesity, which along with increase dental disease, parasitic infestation, and behavioral issues can be avoided with routine preventive care (6). Moreover, early identification and treatment of many diseases can reduce the need for costly interventions later. Clearly, there needs to be an increase in communication between veterinary surgeons and their clients about the advantages of preventive care and this has been recognized by the American Animal Hospital Association and American Veterinary Medical Association who introduced their Preventive Healthcare Guidelines in 2011. Similarly, in 2010 the North American Veterinary Medical Education Consortium recommended that there should be more focus on wellness and disease prevention in veterinary undergraduate education. These measures are very valuable, but Spofford et al. (7) suggest that there is still a need for research “to determine the impact of preventive health care in animals and to distinguish effective preventive health-care services from less effective and ineffective ones.” These authors suggest that there is a need for health—related quality of life (HRQL) evaluations that capture useful information about health that is not readily obtained from the medical case record and that, in particular, generic HRQL instruments that assess wellness could be used to measure the impact of preventive health-care services. In contrast to disease–specific HRQL instruments which have an application limited to sick populations, generic instruments measure the health status of healthy as well as sick subjects and are the only option when co-morbidities exist in the same subject.

Previously, we have reported the development, validation, and reliability of owner–reported generic HRQL instruments (VetMetrica™) for the dog (8, 9) and the cat (10), both of which were generated using data from owners of healthy and sick animals. These are structured questionnaires containing simple behavioral questions for completion online by the owner, with scores generated in four domains of quality of life for the dog (Energetic/Enthusiastic, Happy/Content, Active/Comfortable, and Calm/Relaxed) and three for the cat (Vitality, Comfort, Emotional Wellbeing). In QOL measurement there are two kinds of variables that can be measured—indicator and causal (11). Causal variables like “vomiting” impact the QOL, but don't tell us anything about it, whereas indicator variables like “energy” don't affect it, but do give us information about it. In general, other HRQL tools measure the physical limitations imposed by disease, whereas the VetMetrica™ questionnaires consist only of indicator variables that measure the emotional component of QOL—how the animal “feels.” In this regard, they are unique. The web–based system is compatible with all mobile platforms and since 2014 has been made available to pet owners through the smartphone app PetDialog™ (compatible with iOS and Android devices), developed by Zoetis. PetDialog™ is a white-labeled veterinary clinic branded app designed to facilitate connected care between an owner and a veterinarian. The veterinarian can view the responses from their own clients in a real-time dashboard accessed through a web-platform called VetSupport+. The owner receives timely push-notification alerts to answer HRQL questions within the app where they can also track the results over time. Additionally, the owner, via the app, or the veterinarian, via VetSupport+, can set up medication reminders to assist with compliance of medical recommendations. The PetDialog™ app and Vet Support+ make up the ecosystem that supports 24/7 remote health monitoring of pets.

A key property of any health-related measure is its interpretability, because, without that, the instrument is of little or no practical use when it is used to measure the impact of healthcare interventions. In the medical field interpretability of health measures is an important focus of current research (12), and although there is no consensus amongst our medical colleagues regarding the optimum method of determining interpretability, a number of methods have been described, including relating the HRQL scores to those of a specific population, facilitating judgment as to whether an observed score is typical of what would be expected for that population (norm-based scoring). Norm-based comparisons can be related to the general population, to sub-populations with shared demographics such as age or gender or to a population with a disease (13). Initially, the outputs for VetMetrica™ were reported as “raw” 0–6 scores for each domain, but more recently the calculation of norm-based scores has been reported for the dog tool to improve its interpretability (14). Domain scores are normed to the age—related average healthy dog where the classification is young/middle aged (≤7 years) and old (≥8 years).

While those features which support interpretability of an instrument are fundamentally important, its functionality is markedly enhanced if there is an associated “call to action” which will alert the patient/carer/clinician to the fact that there has been a change in the state of health. Currently in companion animal medicine there is a focus on measuring health status using advanced wearable monitors that can detect changes in activity and a variety of other activities such as drinking, scratching, sleeping, etc. and depending on the monitor will alert the owner to a change in these parameters. These objective measures of physical functions and changes therein may be useful under certain circumstances, especially where changes in mobility or scratching are important indicators of orthopedic or skin disease, and a change in drinking may indicate renal disease or diabetes. However, there are several commonly occurring diseases in animals, such as cancer and obesity that do not exhibit the acute changes in these physical manifestations that will be highlighted with an activity monitor. In these cases, a generic HRQL instrument with an inbuilt health alert would provide useful information for clinician and owner alike.

The aims of this study were firstly to develop an algorithm which will indicate whether a dog is healthy or unhealthy (Phase 1) and incorporate that in the VetMetrica™ dog software to provide a ‘call to action’ to the owner to consult their vet (Phase 2), finally reporting on its use in a large cohort of dogs using the PetDialog™ App (Phase 3).

## Phase 1: Development of the Algorithm Using Raw Domain Scores

### Materials and Methods

All data were retrospective having been collected in a variety of previous studies. All studies were approved by the Ethics Committee of the University of Glasgow Veterinary School and owners gave informed consent for participation in these studies. Dogs (170 ≤ 7 years and 252 ≥ 8 years) were recruited from the University of Glasgow Small Animal Hospital and private veterinary practices. Data used to develop the alert algorithm (Supplementary Information). The health status of all dogs was assessed by the attending veterinary surgeon and the only inclusion criterion for sick dogs was that they were suffering from a chronic condition likely to affect their QOL. There were no exclusion criteria. Owners received an information sheet with the following wording: “*NewMetrica is a small company run by a vet that develops questionnaires to enable us to determine how animals are feeling. We do this by asking you, the owner, certain questions about your dog's behavior. We have developed a short questionnaire called VETMETRICA for this purpose which can be completed online in around five minutes in your own home. We need a large number of owners to complete it so that we can analyze all the information to allow us to test how well the questionnaire works. We would be very grateful for your help with this.”*

Owners completed the Vetmetrica™ HRQL instrument for dogs which contained 22 items, each of which comprised a descriptor (e.g., “active”) with a 7-point Likert rating scale, 0–6 (with 0 meaning “not at all” and 6 meaning “could not be more”), which were used to determine a raw HRQL score (0–6) in each of four domains (Energetic/Enthusiastic, Happy/Content, Active/Comfortable, and Calm/Relaxed) (9, 14). The frequency of assessments for each dog was set by the vet with a minimum period of 2 weeks, however, analysis was restricted to the first assessment for each dog.

Multivariable logistic regression (15) was used to model the relationship between the four HRQL domain scores as covariates or independent variables and the corresponding binary health status of the dog as the response or dependent variable. This was done separately for young/middle aged (≤7 years) and old (≥8 years) dogs (41 sick young/middle aged dogs, 222 sick old dogs, respectively). The statistical model was fit in R (https://www.R-project.org/) using the glmnet package (16). Variable selection was carried out using 10-fold Cross Validation to identify the HRQL domains to be used in each of the models, based on how well they predict the health status of the dogs (17). The performance of the models in predicting the health status of a dog can be evaluated by looking at the sensitivity and specificity. To assess the sensitivity and specificity we must first identify a cut-off value in the regression equation above/below which a dog would be classified as healthy/sick. Varying the cut-off means that different numbers of dogs get a regression score of <0 (which are then classified as sick and the Contact Vet Flag is raised) or more than zero (which are then classified as healthy and no Contact Vet Flag is raised). Sensitivity measures the proportion of positives, i.e., the proportion of sick dogs correctly classified as sick. Specificity measures the proportion of negatives, i.e., the proportion of healthy dogs correctly classified as healthy. The higher the sensitivity and specificity the better however the two measures are dependent and so must be considered jointly. Receiver Operating Characteristic (ROC) scatterplots provide a graphical illustration of the performance over the different cut-offs [by plotting and connecting sensitivity vs. (1-Specificity) values for each cut-off]. The optimum cut-off is the point which is closest to the top left corner of the scatterplot since this represents the point on the ROC curve closest to optimal performance, i.e., maximum sensitivity and specificity).

### Results

**Figure 1:**
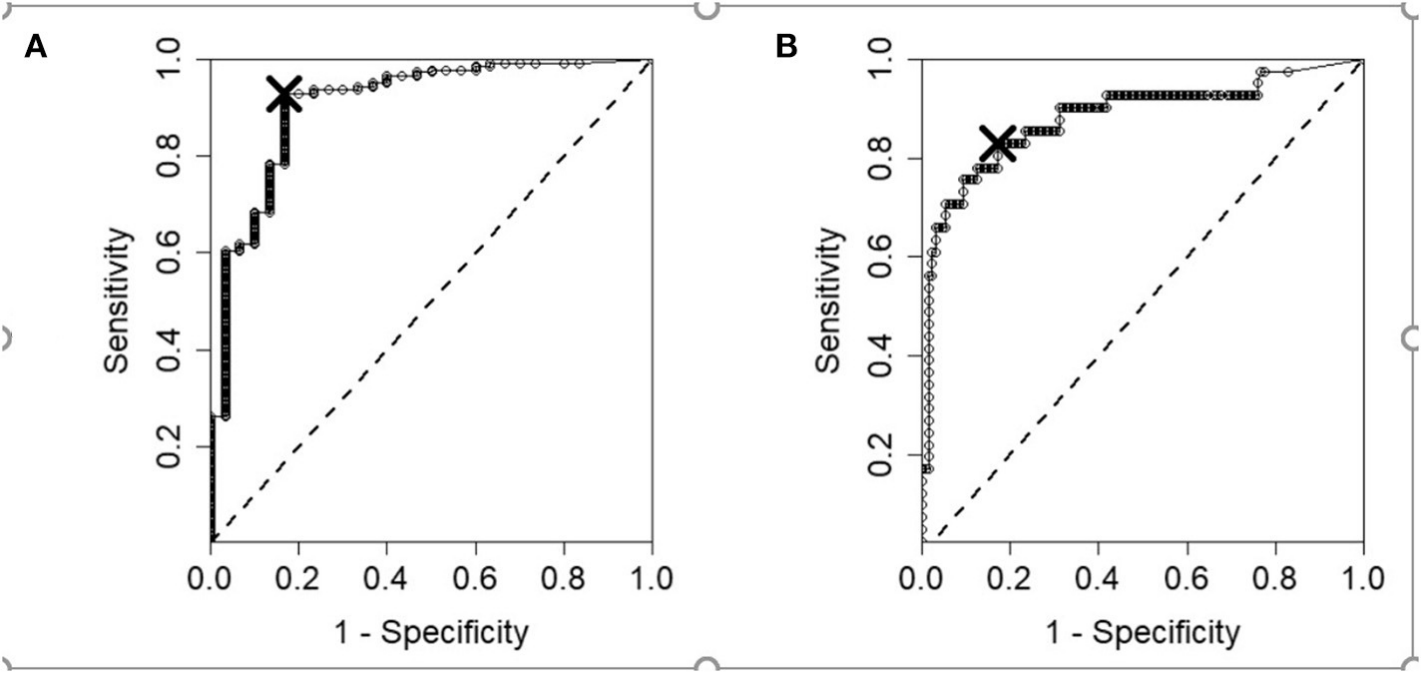
ROC curves showing the results of the health alert model for the **(A)** old and **(B)** young/middle-aged dogs. The cut-offs chosen by the top left corner method are shown as large crosses.

The final Vet Alert models for dogs ≤7 years (young/middle aged) and those ≥8 years (old) both contained the Energetic/Enthusiastic and Active/Comfortable HRQL domain variables, with the model for the young dogs also containing the Happy/Content domain. The results for the Vet Alert models in terms of sensitivity, specificity, and classification accuracy for the old and young/middle aged dogs are shown in Table 1. Figure 1 shows the ROC curves for each of the models, with the sensitivities and specificities from the best cut-off identified by the large X and corresponding to the results in Table 1.

**Table 1:** Sensitivities, specificities, and classification accuracy for the health alert models for old and young/middle-aged dogs.

**Age**	**Sensitivity**	**Specificity**	**Classification Accuracy**
Old	0.93	0.83	0.92
Young/Middle-aged	0.83	0.83	0.83

## Phase 2: Integration of the Algorithm, With a Case Example Included for Illustrative Purposes

### Materials and Methods

The algorithm was embedded in the VetMetrica software by software developers Kyria Ltd. (http://kyria.co.uk/) to create a new output as “Contact Vet Score.” If this score is positive, then no alert is raised (Contact Vet Flag—False). If it is negative then the Contact Vet Flag is raised (Contact Vet Flag—True) and in this case, on completion of the assessment, the owner receives a message to contact their vet.

### Results

**Figure 2:**
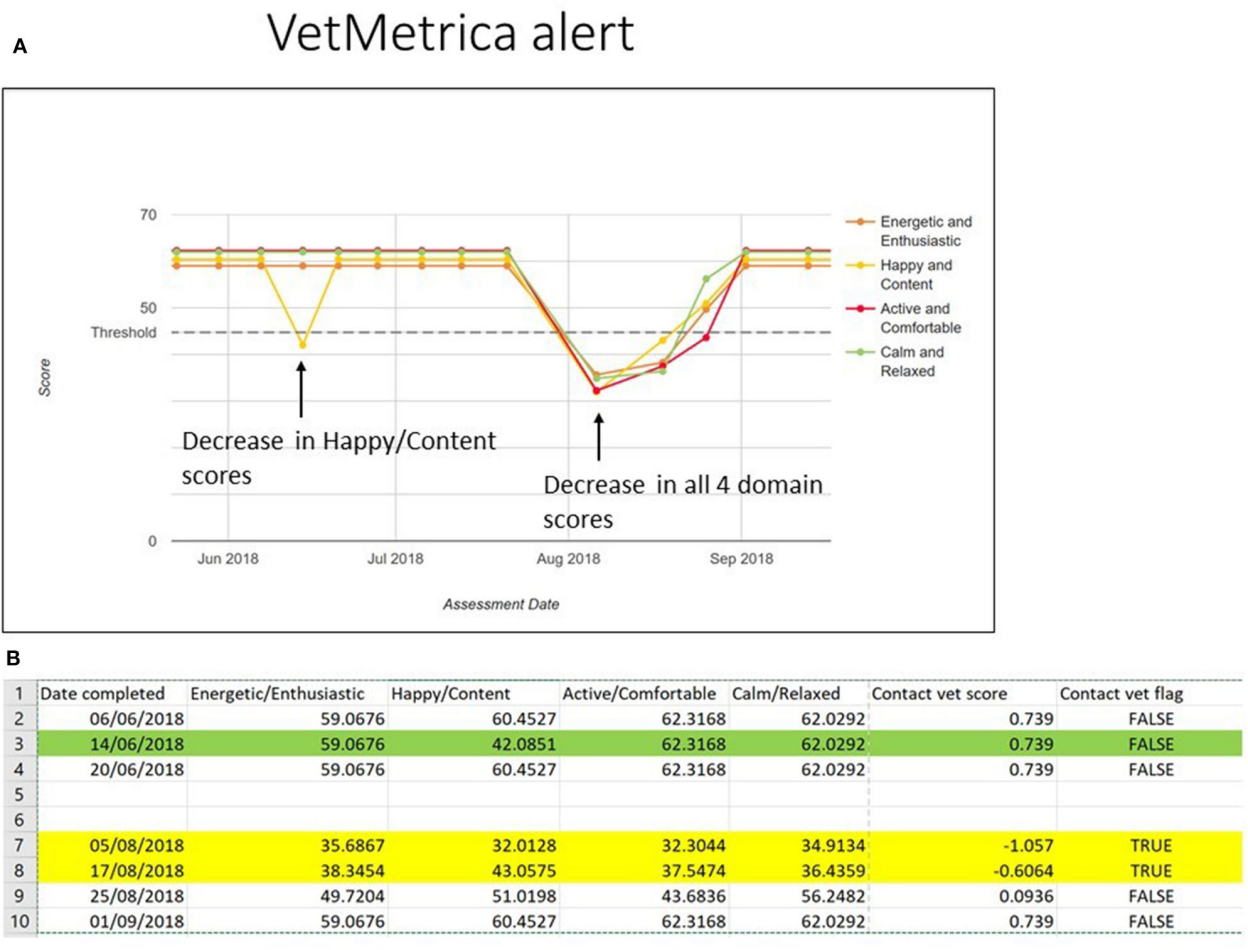
**(A)** A section of the longitudinal results from an 11 year old dog which was suffering from well controlled inflammatory bowel disease (IBD), as they are reported to the vet (normalized scores only). All four domains are individually color coded and normalized HRQL scores are shown on the *y*-axis, where 50 represents the age-related average healthy dog. The dotted line at 44.8 represents the threshold above which 70% of healthy dogs in the appropriate age group will score. **(B)** A the corresponding excerpt from the VetMetrica data extract, with assessment date, domain scores, Contact Vet Score, and Contact Vet Flag. The green highlight refers to the initial decrease in Happy/Content which occurred on the 14th June and the yellow highlight shows the two occasions when the Vet Flag was raised, and an alert was issued to the owner.

Figure 2A shows a section of the longitudinal results from an 11 year old dog which was suffering from well-controlled inflammatory bowel disease (IBD), as they are reported to the vet (normalized scores only). All four domains are individually color coded and normalized HRQL scores are shown on the y axis, where 50 represents the age-related average healthy dog. The dotted line at 44.8 represents the threshold above which 70% of healthy dogs in the appropriate age group will score. HRQL scores were very consistent until mid June when there was a decrease in Happy/Content and again until early to mid-August when all four domain scores showed a considerable decrease, recovering by early September. Because the Vet Flag is raised based on the values of the Energetic/Enthusiastic and Active/Comfortable domains only in dogs ≥8 years, the initial decrease in Happy/Content did not raise the flag.

Figure 2B shows the corresponding excerpt from the VetMetrica data extract, with assessment date, domain scores, Contact Vet Score, and Contact Vet Flag. The green highlight refers to the initial decrease in Happy/Content which occurred on the 14th June and the yellow highlight shows the two occasions when the Vet Flag was raised, and an alert was issued to the owner.

**Figure 3:**
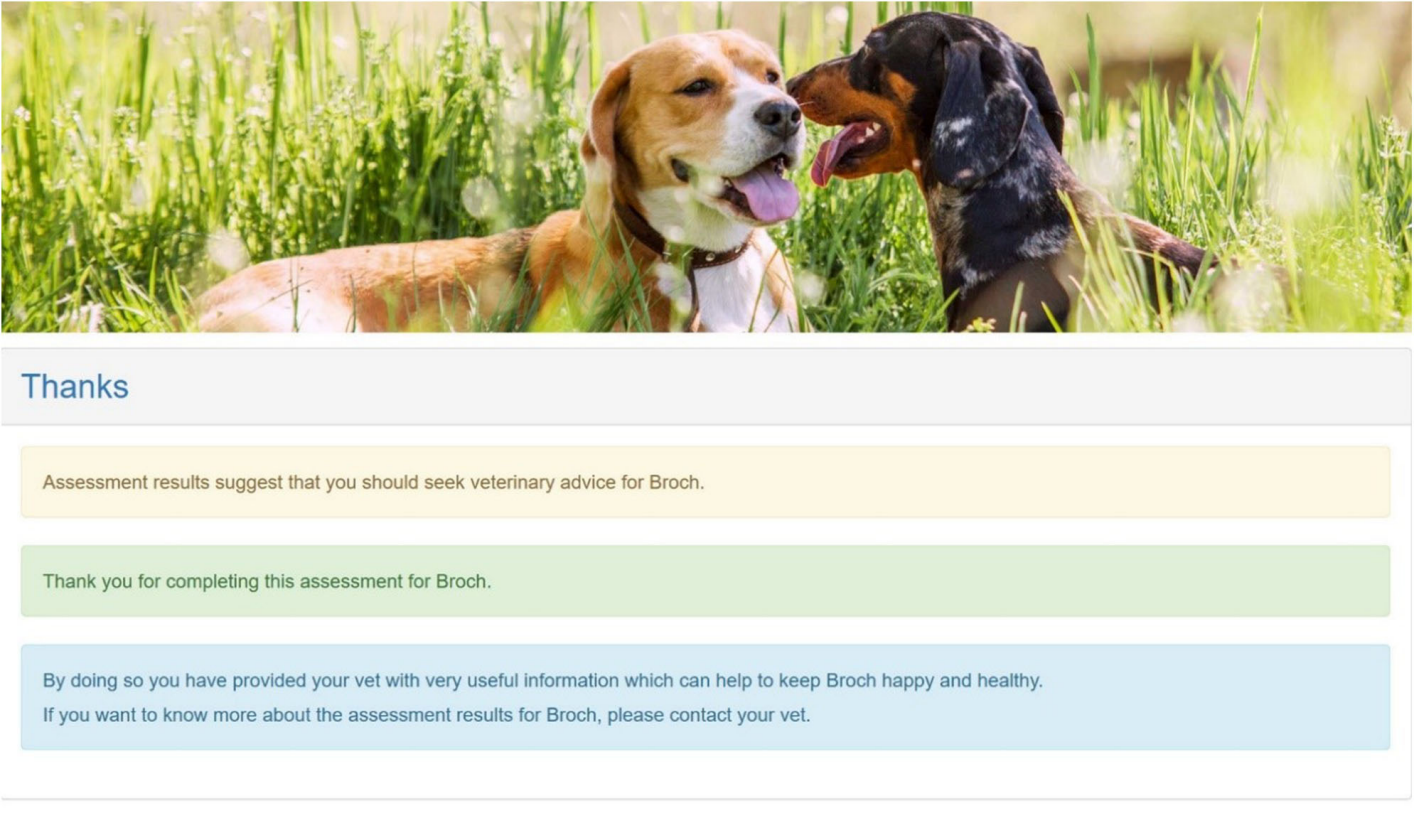
A screenshot of the final “thank you” page of the assessment where the owner is alerted to the fact that they should seek advice from their vet.

Figure 3 is a screenshot of the final “thank you” page of the assessment where the owner is alerted to the fact that they should seek advice from their vet.

## Phase 3 Collection of Data and Reporting of Normalized Scores via an App

### Materials and Methods

Health-related quality of life data were collected from dog owners using VetMetrica™ for dogs via an app (PetDialog™, Zoetis). The VetMetrica™ dog HRQL instrument was incorporated as one of several features in the PetDialog™ app, which also required owners to input their dog's date of birth (DOB), breed, sex, and neutering status. Engagement with the HRQL instrument was entirely at the discretion of owners and uptake was not assessed. The PetDialog™ app was made available to pet owners in the United Kingdom and The Netherlands via veterinary practices and was only accessible using a unique practice code. Due to data protection regulations, the data were anonymized such that the demographic details and geographical location of each dog and owners were unknown. Normalized scores in Energetic/Enthusiastic, Happy/Content, Active/Comfortable, and Calm/Relaxed (14) were reported and the owner's impression of their dog's health status was determined by asking an additional question “Is your dog in perfect health—yes or no” after the 22 questions comprising the assessment were submitted. A definition for perfect health was not included. This question was posed for research purposes only and did not form part of the HRQL assessment. Analysis was restricted to the first assessment for each dog.

### Results

Health-related quality of life data were collected over a period of 72 months (2014–2020) for 6,286 dogs. Data collected via the PetDialog App (Supplementary Information) Data were removed for 178 dogs for which DOB was deemed unreliable. For the remaining 6,018 dogs, there were 2,889 females of which 344 were neutered and 3,219 males of which 354 were neutered. Sixty-eight dog breeds were represented, and these were classified by the developers of the VetMetrica™ system as follows, small (7%), medium 30%), large (33%), and extra large (30%) using a combination of UK Kennel Club breed details and personal experience. Median age was 35 months (Range 1.5–304 months).

**Figure 4:**
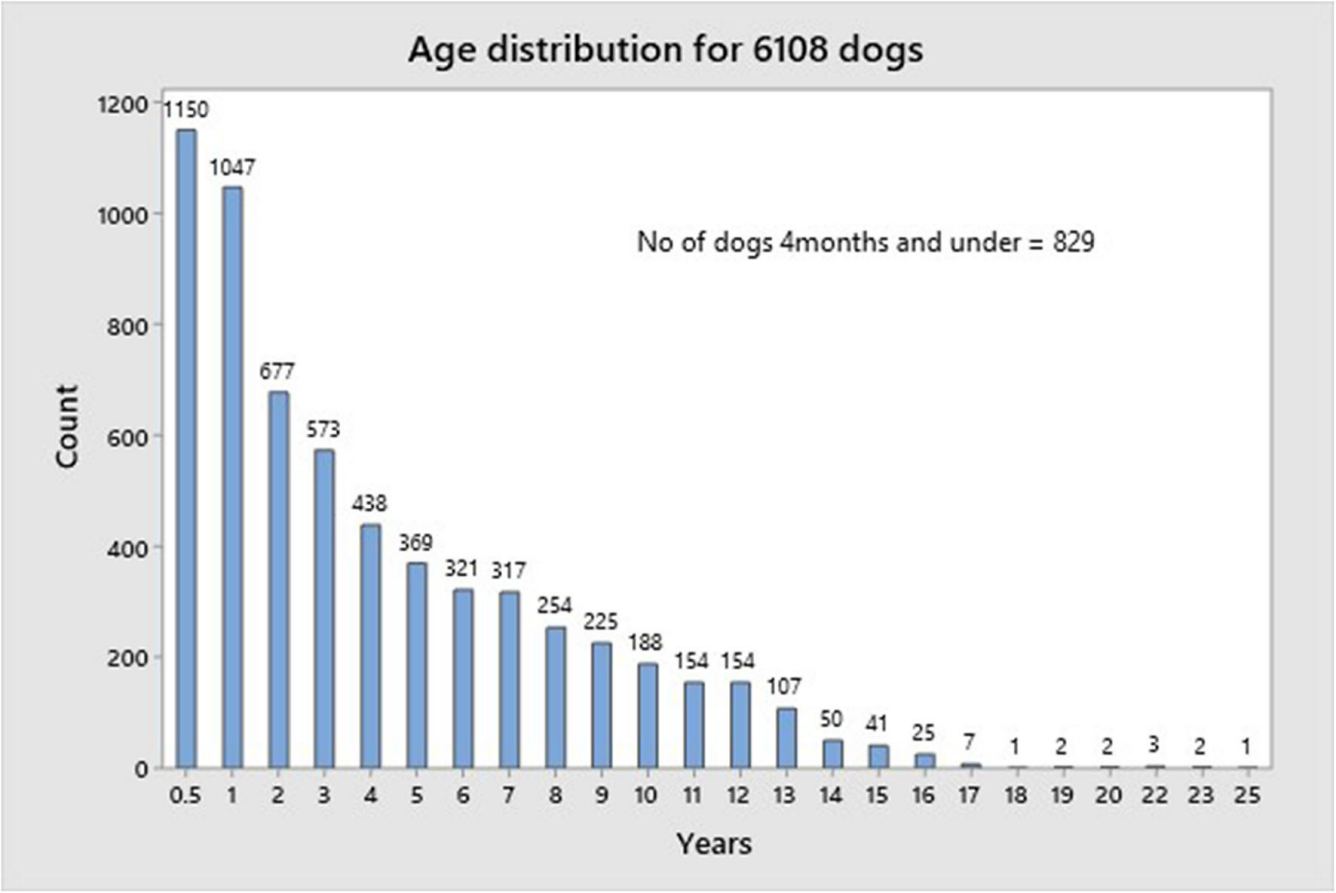
The age distribution of all dogs.

Figure 4 shows the age distribution of all dogs. There is a marked left shift with most dogs fitting into the young/middle age category (≤7 years) (4,892) and of these 1,150 and 1,047 were ≤6 months and ≤1 year, respectively; 1,216 dogs were ≥8 years.

**Figure 5:**
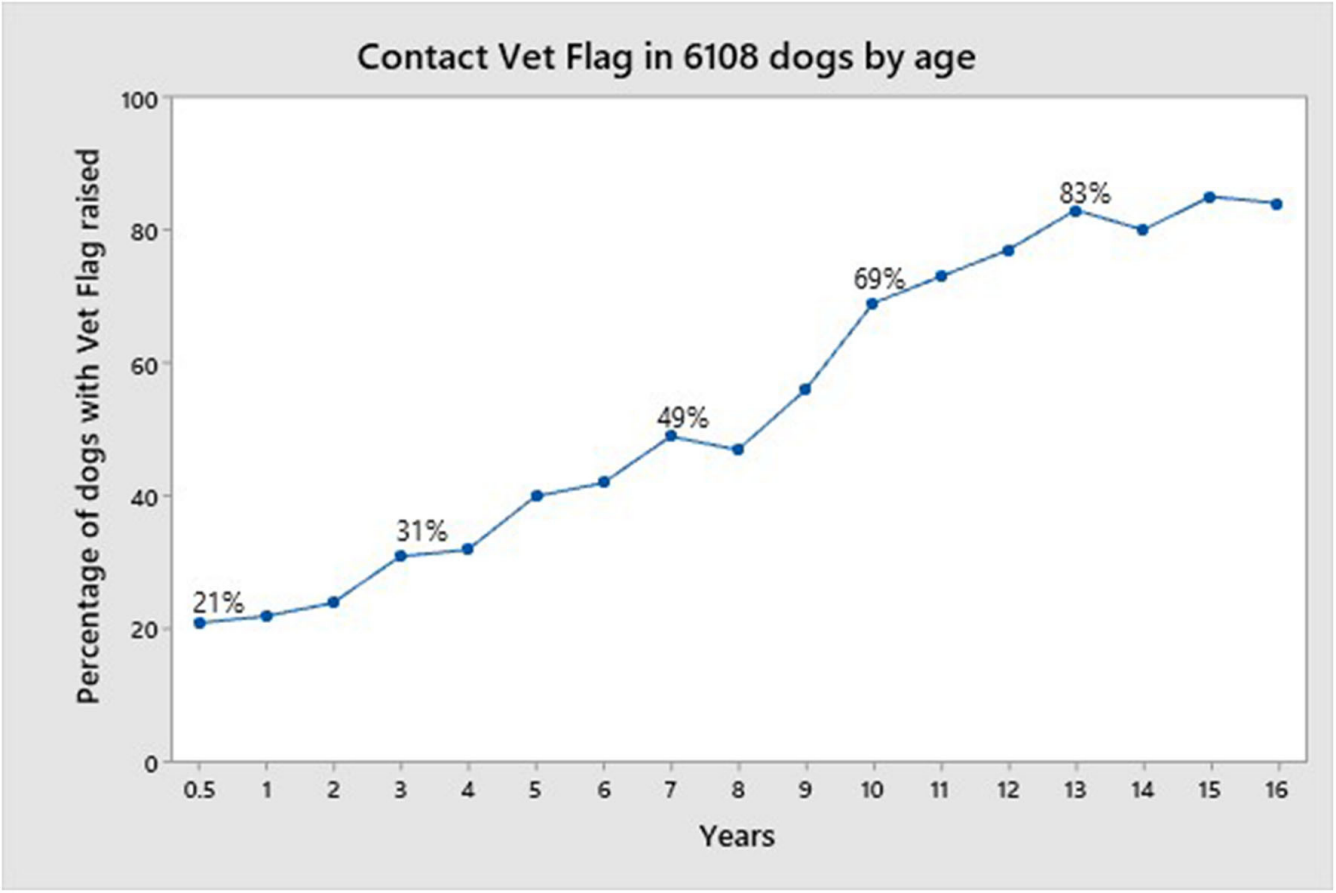
Distribution of the Vet flag by age.

Figure 5 shows the Distribution of the Vet flag by age. The cut-off was 16 years (25 dogs) because in the range 17–25 years there were very few dogs (18 dogs). The Vet flag was triggered for 21% of dogs aged between 6 weeks and 6 months and thereafter there was a rise in the number of dogs for whom the flag was triggered, 31, 49, 69, and 83% for ages 4, 8, 11, and 14, respectively, with an increase in the rate of rise from 8 years.

**Figure 6:**
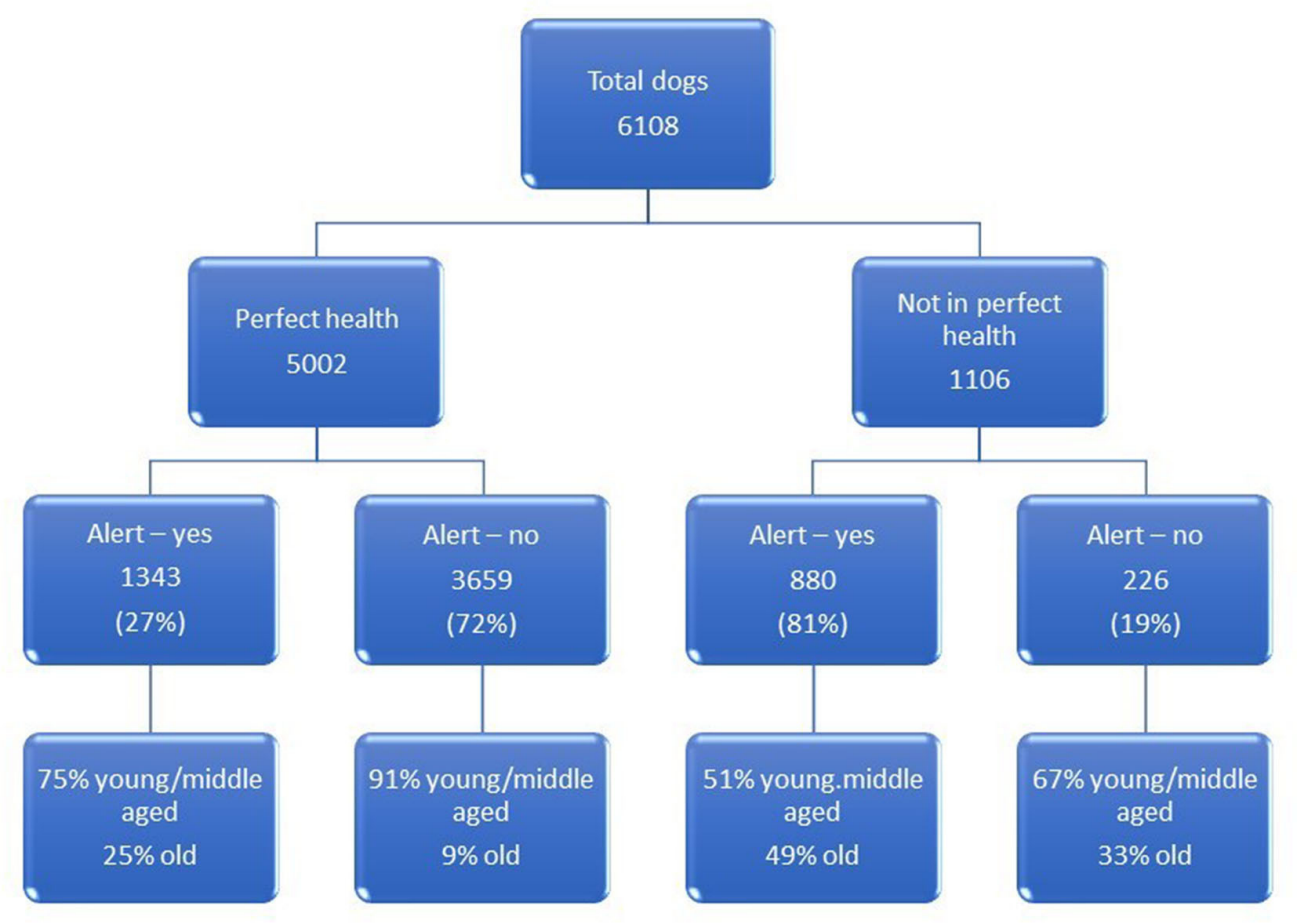
An overview of the owners' impression of health status for the 6,108 dogs along with details of their age category and whether the Vet flag was triggered.

Figure 6 gives an overview of the owners' impression of health status for the 6,108 dogs along with details of their age category and whether the Vet flag was triggered. Of the 5,002 dogs considered to be in perfect health by their owners the Vet flag was triggered for 1,343, indicating that they were not in perfect health according to their HRQL profile of scores. Of these dogs 75% were classified as young/middle aged and 25% were old.

## Discussion

Two vet alert models, one for young/middle aged dogs (≤7 years) and one for old dogs (≥ 8 years), were developed to identify whether dogs were sick or healthy based on four HRQL domain scores derived via an online instrument for monitoring HRQL in dogs (9). The models were created using the elastic net multivariable logistic regression with all four HRQL domain scores simultaneously included as potential variables. The elastic net method chooses which variables go into each model, with the aim of avoiding the inclusion of variables that are not predictive of the health status of the dogs. This, along with using 10-fold cross validation procedure to choose the penalty parameter, improves how well the models perform in dogs that were not part of the original data used to train the model, making the alert system more suitable for use as an early warning system outside of this initial dataset.

The variables chosen by each of the models indicated which of the HRQL domain scores were most predictive of health status. Both the Energetic/Enthusiastic and Active/Comfortable HRQL domains were included in both models because they helped to differentiate between healthy and sick dogs in both age groups. The Happy/Content HRQL domain was only included in the model for young dogs which suggests that based on current data this HRQL domain only helps determine the health status of young dogs. It is unclear why this might be the case. It may simply be that there is not enough data to distinguish the complex relationship between the validated measures of HRQL and a veterinary diagnosis of sickness. The high correlations Happy/Content has with Energetic/Enthusiastic and Active/Comfortable within the model indicate that this may be the case, and it is these high correlations that result in Happy/Content being removed by the elastic net model's variable selection procedure. Finally, both vet alert models did not contain the Calm/Relaxed HRQL domain score. The Calm/Relaxed HRQL domain has previously been shown to be less consistent in differentiating health status compared to other domains, and this has previously been attributed to the fact that this domain may reflect stable personality traits which are less sensitive to changes in health status (9, 14). The lack of all domains in the model does not necessarily mean that each of the HRQL domains is not reflective of HRQL, but rather that a combination of them is needed to predict a veterinary diagnosis of sickness. While it may seem logical to include all the validated HRQL domains regardless, it would be statistically inappropriate to do so as this could result in worse predictive quality when the model is applied to a general dog population.

In terms of sensitivity and specificity, both vet alert models performed well, with the results for both models being close to the perfect score of 1 (Table 1 and Figure 1). This meant that the models were able to identify 93 and 83% of sick dogs respectively for young/middle aged and old subjects within the data set. Similarly, they correctly predicted the health status of 83% of healthy dogs for both young/middle-aged and old dogs. While these sensitivities and specificities are only reflective of the performance of the models within the dogs selected for the study, the model selection procedure which included cross-validation for selection of the variables and an elastic net regression coefficient estimation method, should ensure that similar performance will be obtained for other cohorts of dogs. Indeed, the case shown in Figures 2A,B of the dog with IBD provides evidence for the soundness of the alert. When only one domain score deteriorated (Happy/Content), which in the case of old dogs is not included in the alert algorithm, and besides which could have been the result of conditions other than health, there was no alert triggered, but when there was a general deterioration in scores, coinciding with a flare-up of the clinical condition, the alarm was raised.

The majority of dogs for whom there were HRQL data recorded were in the young/middle aged category with a large proportion of these aged 1 year and under and this may have accounted in part for the high proportion of entire dogs. Furthermore, ~20% of this age group triggered the vet flag. While this is understandable given that some of these dogs might have had an acute illness or something as simple as a cut pad that would affect their mobility and hence their HRQL scores on the day of assessment, it is notable that 38% of these dogs were 4 months and under, and so such an explanation is less likely. The behavior patterns of young puppies differ from that of the adult and as the algorithm for the vet flag was derived using a population of dogs aged 6 months and over, this may account for the unexpected incidence of the Vet flag triggering in this group of very young dogs. Other than for the very young dogs, the incidence of vet flag triggering was as expected with a steady rise as age increased. From 1 to 7 years of age there was a 28% rise and then in a similar period from 8 to 14 years the rise was 34%. Thereafter the rate of vet flag was static at around 83%. This accelerated incidence from 8 years on concurs with the increase of chronic disease in geriatric populations, with a consequent decrease in QOL.

In people, wellness is often seen as a critical concept for understanding preventive aspects of disease, disability, and social breakdown and it has been the focus of much research relating to the support of the elderly living independently at home (18). To the authors' knowledge there is no such wellness monitoring available for people throughout their life cycle. However, the shorter lifespan of companion animals affords the opportunity to measure wellness using generic HRQL measures throughout life and has distinct advantages in terms of early disease detection, monitoring the efficacy of therapeutic interventions, and providing humane endpoints for individuals at end of life.

Currently, pet health care plan providers assure owners that by subscribing to a wellness health plan they are ensuring the best preventative health care for their pet. Other than vaccination and routine ectoparasite treatment, these generally include an annual or biannual veterinary examination with or without additional veterinary nurse consultations. However, between these visits the Vet is reliant on the owner to make an appointment if their pet seems to be unwell. It has been shown here that owners frequently miss the signs of ill health in their animals. The Vet flag was triggered in 1,343 (27%) of 5,002 dogs deemed healthy by their owners, and this compares well with statistics published for the cat (26%) (10). Of these 1,343 dogs, 75% were young/middle aged and 25% were old. Much anecdotal evidence exists to suggest that owners frequently confuse the signs of illness in the elderly animal with “just getting old,” but this does not explain the high proportion of dogs that were in the young/middle aged category. Since this initial assessment may have coincided with an acute disease episode or a traumatic event, these cannot be ruled out as a cause of low scores in HRQL, but such dogs are unlikely to be deemed in perfect health by their owners. Accordingly, we may assume that the majority might have been suffering from an underlying disease process like osteoarthritis. Osteoarthritis very often begins in early life, but the clinical signs may not manifest themselves until the animal is older, or until these signs are highlighted through the use of a Health Risk Assessment administered routinely by the veterinary practice (19). If lifestyle changes/treatment are instigated early this can mitigate progression of the disease (7). Accordingly, the incorporation of a generic HRQL monitoring tool into existing wellness plans for companion animals would bridge the gap between scheduled veterinary visits. However, wellness plans involve a financial commitment for the owners which may limit their uptake to those who have the necessary resources, especially if the owner has multiple pets. Currently, to the authors' knowledge, there is no provision for wellness monitoring within any veterinary health plan. The work described here has demonstrated that the PetDialog™ app and Vet Support+ ecosystem, which supports 24/7 remote health monitoring of pets, is an efficient way for vets to provide all their owners with the opportunity to monitor their animal's wellness throughout their lifetime, providing the vet with a mechanism to identify health problems early via the vet flag and most importantly to stimulate owners to be more proactive in seeking veterinary attention. However, it was a major limitation of this study that there was no available mechanism to determine how many owners responded to the vet flag and did subsequently consult their vet. Establishing this will be important moving forward and it may be that it will be necessary to make more explicit the message that the owner receives. When the “thank you” page was designed the research team did not want the alert message for the owner to be too alarmist and so it errs on the gentle side.

It is encouraging to note that most dogs were registered for PetDialog when they were young, perhaps indicating that owners were prepared to use the app rather like a wellness plan. However, this would depend on continued use of the tool and experience with human healthcare apps has shown that to be effective continuous use of mobile Health apps is vital. Further research into the longitudinal use of VetMetrica™ within PetDialog™ is being undertaken to establish if owners are engaged in a sustainable fashion and this will be the focus of a further publication.

## Data Availability Statement

All datasets generated for this study are included in the article/Supplementary Material.

## Author Contributions

Conceptualization: MW-O, JR, VD, and ES. Provision of data: JR and AW. Statistical analysis: VD and ES. Algorithm: VD, MW-O, and JR; App development: JR and MW-O. Writing, review, and editing: JR, VD, ES, MW-O, and AW. All authors contributed to the article and approved the submitted version.

## Conflict of Interest

JR is a Director in NewMetrica Research and AW is a Director in Outcomes Research International, Zoetis. The remaining authors declare that the research was conducted in the absence of any commercial or financial relationships that could be construed as a potential conflict of interest.
