# Corrigendum: Development of an Early Warning System for Owners Using a Validated Health-Related Quality of Life (HRQL) Instrument for Companion Animals and Its Use in a Large Cohort of Dogs

**DOI:** 10.3389/fvets.2021.676049

**Published:** 2021-04-15

**Authors:** Vinny Davies, E. Marian Scott, M. Lesley Wiseman-Orr, Andrea K. Wright, Jacqueline Reid

**Affiliations:** ^1^School of Computing Science, University of Glasgow, Glasgow, United Kingdom; ^2^School of Mathematics and Statistics, University of Glasgow, Glasgow, United Kingdom; ^3^School of Education, University of Glasgow, Glasgow, United Kingdom; ^4^Outcomes Research, International Centre of Excellence, Zoetis, Dublin, Ireland; ^5^NewMetrica Ltd., Glasgow, United Kingdom; ^6^School of Veterinary Medicine, University of Glasgow, Glasgow, United Kingdom

**Keywords:** Health-Related Quality of Life (HRQL), owner questionnaire, smartphone app, dogs, early warning system, wellness, preventive medicine

M. Lesley Wiseman-Orr was not included as an author in the published article. The published research was divided into two parts — an “alert” (which was intended as an initial early warning system for dog owners) development phase for an existing health-related quality of life (HRQL) tool and some 5 years later the reporting of use of the alert in a large cohort of dogs, which was the main focus of the paper. Although Dr. Wiseman-Orr was not involved in the second part of the study, she was involved in the conceptualization of the study, and in the development of the alert and the app. The original authors now appreciate that omitting her from the author list was a most unfortunate oversight on their part and wish to remedy that if possible.

The corrected Author Contributions Statement appears below.

Conceptualization: MW-O, JR, VD, and ES. Provision of data: JR and AW. Statistical analysis: VD and ES. Algorithm: VD, MW-O, and JR; App development: JR and MW-O. Writing, review, and editing: JR, VD, ES, MW-O, and AW. All authors contributed to the article and approved the submitted version.

The authors apologize for these errors and state that they do not change the scientific conclusions of the article in any way. The original article has been updated.

